# Potential smart grid vulnerabilities to cyber attacks: Current threats and existing mitigation strategies

**DOI:** 10.1016/j.heliyon.2024.e37980

**Published:** 2024-09-16

**Authors:** Bishowjit Paul, Auvizit Sarker, Sarafat Hussain Abhi, Sajal Kumar Das, Md. Firoj Ali, Md Manirul Islam, Md. Robiul Islam, Sumaya Ishrat Moyeen, Md. Faisal Rahman Badal, Md. Hafiz Ahamed, Subrata Kumar Sarker, Prangon Das, Md. Mehedi Hasan, Nazmus Saqib

**Affiliations:** a Department of Mechatronics Engineering, Rajshahi University of Engineering & Technology, Rajshahi, Bangladesh; b Department of Electrical and Electronic Engineering, Daffodil International University, Dhaka, Bangladesh

**Keywords:** Smart grid, Cyber physical system, Cyber attack, Detection, Mitigation

## Abstract

A novel concept in the realm of conventional electricity grids, known as the “smart grid,” has emerged to explore the most effective methods for integrating green and renewable energy sources. By leveraging existing technologies for its communication network, the Smart Grid also inherits their associated drawbacks. Exploiting these vulnerabilities can lead to severe consequences such as privacy breaches, cascading failures, or even system-wide blackouts. Securing the Smart Grid is now paramount to ensuring its optimal performance. This document aims to provide a comprehensive analysis of the Smart Grid. We begin by examining its inherent weaknesses, followed by a classification of common attacks and their potential impacts. Subse-quently, we delve into strategies for mitigating and detecting these attacks, utilizing appropriate algorithms. Lastly, we address current research challenges and propose future initiatives aimed at enhancing cybersecurity measures to safeguard smart grids from cyberattacks. Moreover, this review emphasizes the intricate relationship between technological vulnerabilities and cybersecurity challenges within the Smart Grid framework. It offers a nuanced perspective that highlights specific areas requiring heightened attention to establish an effective and robust defense against potential threats.

## 1 Introduction

A communication network is integrated with the electricity distribution system to form a modern smart grid, an infrastructure of a complex cyber-physical power system enabling bidirectional power and information transfer [1,2]. By 2023, 65 % of electrical firms are expected to have invested in flexibility services, potentially reaching up to 35 % of installed capacity [3]. “Smart Grid” is a prevalent term in electric utility jargon [4], leveraging computer-based automation and remote control [5] to enhance effectiveness, reliability, economy, and sustainability of energy generation and delivery [6]. However, smart grids, being computerized remote-control systems overseeing electricity distribution, are vulnerable to cyberattacks. Cybercriminals deliberately target them to disrupt operations or gain unauthorized access to the system, posing risks such as significant outages and financial losses. Successful cyberattacks can compromise private information or even gain full control of the system, manipulating power flows or disrupting operations. The severity of such attacks depends on their sophistication and the effectiveness of security measures in place.

As hackers and fraudsters continually exploit new technologies to infiltrate networks and compromise data [7], understanding smart grid vulnerabilities and implementing effective mitigation strategies becomes paramount. This paper aims to conduct a comprehensive analysis of existing threats to smart grids and explore diverse mitigation strategies to enhance their cybersecurity. Furthermore, it examines current research advancements and identifies critical gaps that need addressing to ensure the safety and security of smart grid systems. Addressing these gaps will bolster our ability to ensure the resilience and protection of modern smart grid infrastructures.

Recent sporadic cybersecurity incidents worldwide have exposed vulnerabilities in smart grids, underscoring the urgent need for robust cybersecurity measures, as detailed in Table 1. As technology becomes more integrated into daily life and cyber-physical systems grow more intricate, the risk of smart grid cyber attacks escalates. Collaboration among governments, utility companies, and cybersecurity specialists is essential to implementing preventive measures that mitigate these risks and uphold the reliability and safety of our power systems.

**Table 1:** Several sporadic malicious and unintentional real occurrence of cyber security event.

Incident Title	Date	Incident
Davis-Besse Shutdown	January 2003	For maintenance, the Davis-Besse nuclear power station in Ohio was shut down. The automatic safety monitoring system became unusable due to the Slammer worm [8,9].
Hatch Nuclear Facility Shutdown	March 2008	The emergency 48-h shutdown of the Nuclear Power Station (Hatch) in the vicinity of Baxley, Georgia, was brought on by a software upgrade that was installed on a single computer [8]
Stuxnet Worm	July 2010	The Stuxnet worm, which was first identified, first attacked Iranian uranium enrichment plants before spreading to other nations, is the earliest known instance of malware [10,11]
Saudi Aramco Systems Interruption	2012	Saudi Aramco, A biggest oil corporation in the global and a Saudi Arabian enterprise, had its systems interrupted by the Shamoon ransomware [12]
Ukrainian Power Grid Attack	2015	The attack on the electricity grid in Ukraine, which caused blackouts, was timed perfectly for the electric grid [13].
Russian Hackers Power Grid Break-in	2016	Russian hackers broke into a northern Ukrainian electrical grid during Christmas season. They compromised an data network which is IT based and brought about OT (auto- matic control system) issues in the substations that occurred several hours of power interruptions [14].
US Power Utilities Spear-phishing Attack	March 2018	Russian hackers gained access to the control systems of US power utilities through spear-phishing attacks on employees, compromising the security of the grid. While no outages were reported, the incident raised concerns about the vulnerability of US infrastructure to cyber attacks [15].
Cyber Espionage Campaign	2017–2018	Russian hackers carried out a cyber espionage campaign targeting energy companies in Europe and the US, using social engineering and spear-phishing techniques to gain access to sensitive information about industrial control systems. The attack is believed to be laying the groundwork for future attacks [16].
Petrochemical Facility Malware Attack	2017–2018	A malware attack on a petrochemical facility in Saudi Arabia targeted the facility's safety systems and was designed to manipulate control systems of industrial to harm the physical world. This highlights the potential for cyber attacks to have real-world consequences beyond data theft or disruption [17].
Water Treatment Plant Hack Attempt	February 2021	Remote attackers successfully obtained unauthorized re-mote access to the control system of a water treatment facility in Florida. Their malicious intent was to manipulate the water supply by elevating the levels of sodium hydroxide (lye) to hazardous levels [18].
SolarWinds Supply Chain Attack	2020	A cyberattack known as the SolarWinds supply chain assault was found in December 2020. An update that was provided to clients by the network and device management software firm SolarWinds contained malware that may have compromised thousands of networks [19].

The smart grid represents a modern evolution in electrical infrastructure, facilitating bi-directional information and power flow within a sophisticated, automated, and distributed energy delivery network. It enhances efficiency and reliability, supports the integration of renewable energy sources and the proliferation of electric vehicles, offers consumers new tools to optimize electricity consumption, and contributes to reducing carbon emissions.

The Table 2 below outlines the key differences between traditional grids and smart grids:

**Table 2:** Difference between traditional grid and smart grid.

Comparative Dimension	Traditional Grid	Smart Grid
Genre	Electro mechanical grid	Digital grid
Concept	Ancient	First official definition is given in 2007, so newly introduced
Generation	Centralized	Distributed
Communication and Control	One-way communication, no feedback or con-trol	Two-way communication, real-time monitoring and control
Energy Efficiency	Limited control over energy usage and distribu-tion, less efficient	Advanced control over energy usage and distri-bution, more efficient
Renewable Energy Integration	Limited sources	Advanced sources, optimized use
Energy Storage	Limited capacity	Advanced systems, optimized energy usage
Grid Reliability	Less reliable, prone to grid failures and power outages	More reliable, less prone owing to grid failures and outages
Maintenance and Repair	Reactive maintenance, requires manual inspec-tion and repair	Proactive maintenance, uses sensors and pre-dictive analytics for maintenance and repair
Cybersecurity	Less vulnerable to cyber attacks, limited digital communication and control	More vulnerable to cyber attacks, requires ad-vanced security measures
Cost	Lower initial cost, but higher operational cost	Higher initial cost, but lower operational cost in the long run
Sustainability	Limited sustainability, relies heavily on fossil fuels	More sustainable, promotes the use of renew-able energy and reduces carbon footprint

In general, there are significant distinctions between conventional and smart grid technologies, encompassing differences in design, infrastructure, and capabilities. The concept of the smart grid first emerged in the United States in the early 2000s, with the Department of Energy launching the Smart Grid Initiative in 2007 to accelerate technological development and deployment [6]. Since then, countries like Germany, China, and South Korea have also invested heavily in smart grid infrastructure.

The choice between traditional grid technology and smart grid technology hinges on various factors, including specific energy system requirements, existing infrastructure, and regulatory frameworks. Smart grid technology is particularly suited for environments requiring more effective and reliable energy allocation and consumption, such as areas with high energy demand or frequent power outages. Additionally, smart grid technology proves advantageous in regions with significant potential for renewable energy generation, such as solar and wind power, due to its ability to integrate intermittent energy sources into the electricity grid [20].

This paper aims to categorize various cyber-attacks targeting smart grids, as shown in Fig. 2. To achieve this goal, the paper employs techniques like machine learning, deep learning, and graph signals to systematically develop detection methods for these attacks. Subsequently, the paper explores different defense and mitigation strategies, including algorithmic and architectural approaches. Additionally, it proposes a proactive self-mitigation strategy designed to preemptively address system vulnerabilities before potential exploitation by attackers. The paper concludes by discussing future developments that could mitigate the impact of cyber-attacks on smart grids.

The primary focus of this article is to provide a comprehensive analysis of cyber-attacks on smart grids, detailing the detection, defense, and mitigation techniques employed. Through this analysis, the paper aims to deepen understanding of the cybersecurity landscape within smart grids, advocating for stronger security measures to protect these critical systems from cyber risks. The paper also proposes solutions to enhance smart grid security, thereby reducing the likelihood and impact of cyber-attacks.

**Fig. 1:**
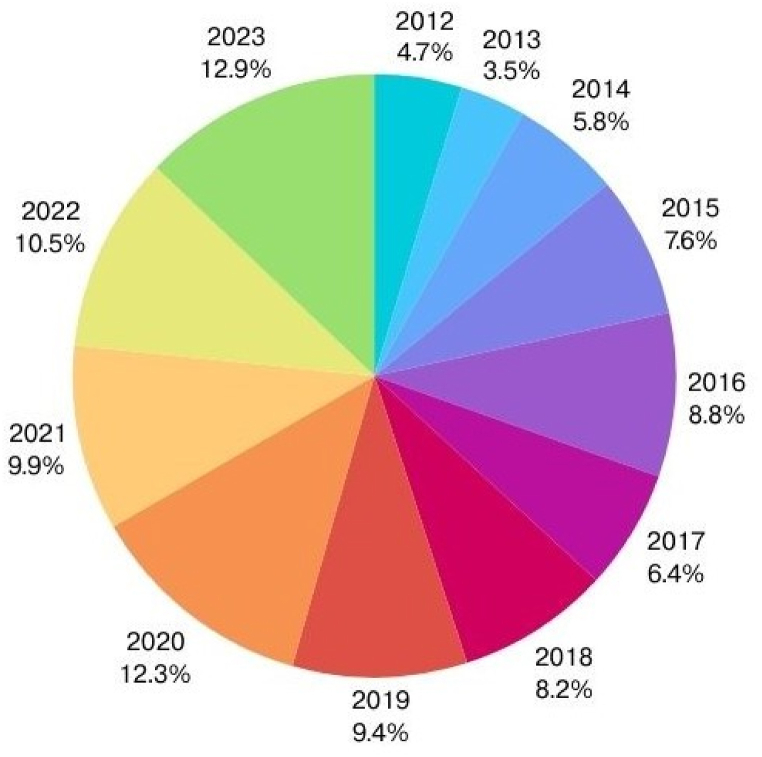
Year wise distribution of paper related to smart grid.

Fig. 1 serves as an insightful visualization derived from a meticulous selection process of data focused on detection and defense techniques specific to vulnerabilities within smart grid (SG) networks. While our dataset may not encompass every publication on this topic, we have diligently curated research that significantly contributes to understanding and mitigating SG vulnerabilities.

**Fig. 2:**
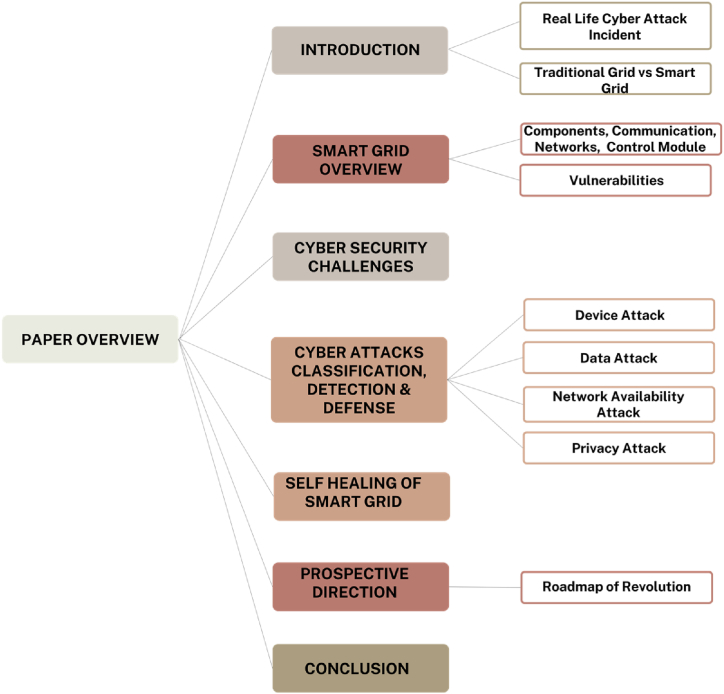
Overview of the paper's structure and main components.

The illustration delineates the evolving landscape of research trends in smart grid vulnerability analysis, showcasing a discernible surge in activity from 2012 to 2016, reflective of an escalated recognition of cybersecurity imperatives. While a marginal downturn in 2013 hints at potential shifts in prioritization, ensuing years witnessed a gradual wane in research focus on SG vulnerabilities. However, the notable resurgence in 2020, propelled by emergent threats and technological advancements, reignited scholarly interest. Despite a slight regression in 2023, the overarching trajectory underscores persistent endeavors to confront cybersecurity challenges within SG networks through pioneering research and innovative solutions.

This review paper critically compares recent studies on the cybersecurity of smart grids, emphasizing the crucial need to secure these systems against cyber attacks. The selected studies present different approaches for detecting and mitigating attacks, and the review analyzes their methodologies, techniques, and results. Common vulnerabilities in smart grid systems are identified, and the effectiveness of various detection and mitigation techniques, including machine learning and anomaly detection, is evaluated. The paper concludes with a comparison table summarizing the key findings of each study, highlighting the ongoing development of effective cybersecurity measures for smart grids. Table 3 serves as a comprehensive repository of comparative insights drawn from recent scholarly investigations into the cybersecurity domain of smart grid networks. It meticulously dissects each study based on a multifaceted evaluation framework, encompassing the spectrum of cyber threats studied, the methodologies employed for detection and mitigation, and the diverse performance metrics used for evaluation.

**Table 3:** Comparison of recent papers on smart grids: Key findings and contributions.

Reference	Proposed	Findings	Limitations	Attack descrip- tion	Defects	Class	Detect	Defense
Yan et al. 2012	Cyber security for communications on the smart grid	The idea of comprehensive solution and communication architecture.	No specific way to find out problem of solution.	✓	✓	✗	✗	✓
Amin & Massoud 2012	Smart grid secu-rity, privacy, and resilient architec- tures: Opportuni- ties and challenges	basic principle about security and obstacles.	Architectural or any algorithm based solution was not mentioned.	✓	✓	✗	✓	✗
Pandey et al., 2016	Threats to cyber security in the Smart grid framework	Infrastructure framework with deep research dirrection	Attack detection or defense method was not discussed.	✓	✓	✗	✗	✗
Kotut and Wahsheh 2016	Security challenges, some method and techniques to improve in future.	Some prospective solutions in miti- gating attacks in efficient way.	Attacks are not properly classified and detection mehods are missing.	✓	✓	✗	✗	✓
Weerakkody and Sinopoly 2019	Proposed research goals a with nec- essary framework and approached to bridge the gap in cyber security.	It gives some method which can detect attacks and also gives the mitigation method.	Attacks are not well classified and vulnerabilities were not discussed properly.	✓	✗	✗	✓	✓
Mohammadi & Fazel 2021	Emerging challenges in smart grid cybersecurity enhancement	three mitigation and detection technique of FDIA	Focused about data attack only but not all of the attack was included.	✓	✗	✗	✓	✓
Zhang et al., 2021	cyber-physical at-tack on smart grid and defense	Vulnerabilities, various attack, moving target defense, watermarking.	classification, attack detection model, other defense technique that could be used.	✓	✓	✗	✗	✓
The Proposed Paper	Different frameworks for addressing , detecting, mitigating cyber attacks.	Proper attack descriptions, challenges, classification, and methods for identifying and make protection against attacks.	Some techniques lack empirical validation, while others may become outdated. Additionally, new advanced techniques are introduced.	✓	✓	✓	✓	✓

By critically assessing the strengths and weaknesses of each study, the table not only sheds light on the intricacies of smart grid cybersecurity but also unveils recurring challenges and vulnerabilities embedded within these critical infrastructures. This meticulous analysis empowers researchers, practitioners, and policymakers to discern emerging trends, identify knowledge gaps, and pinpoint areas ripe for further exploration and innovation.

Furthermore, the synthesized findings presented in Table 3 serve as a guiding beacon for developing tailored cybersecurity strategies and technologies aimed at bolstering the resilience and reliability of smart grid ecosystems. In an era marked by escalating cyber threats and rapid digitization, the insights encapsulated within this table are instrumental in shaping the future trajectory of research, policy formulation, and industry practices focused on safeguarding the integrity and security of smart grid networks worldwide.

In summary, this paper examines various approaches and methodologies for detecting and mitigating cybersecurity threats within smart grid systems. It recognizes the benefits of smart grid technology, such as enhanced energy efficiency and reliability, while also addressing the challenges arising from increased complexity and interconnectivity. The primary goal is to identify and implement effective cybersecurity measures to safeguard critical infrastructure and ensure the safety and security of individuals.

## 2 Smart grid overview

**Fig. 3:**
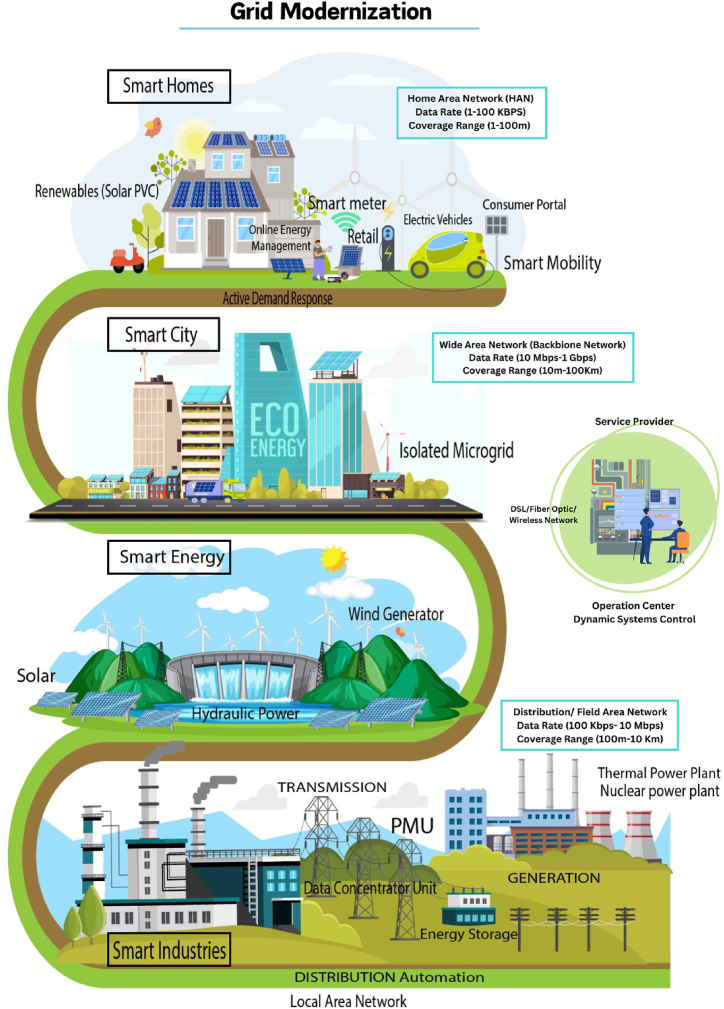
Smart grid (traditional electric power grid from an electro-mechanically controlled system to an electronically controlled network).

With the global increase in electricity demand, there is a continuous need to add more generation capacity to our power systems. However, this often involves the addition of coal-fired thermal power plants, which contribute significantly to carbon emissions. In today's world, there is a strong focus on adopting environmentally friendly and sustainable energy solutions. This can be achieved by incorporating natural-based renewable energy sources like photovoltaic (PV), sun, and wind power into our existing energy systems. By doing so, we can reduce pollution levels, minimize carbon footprints, and promote the use of green energy worldwide. To address the challenge of reducing carbon dioxide emissions while meeting the growing power demand, it is essential to integrate renewable sources into the current power grid. This integration will result in an efficient, cost-effective, and sustainable energy system known as a smart grid. The Fig. 3 represents the entire structure of the smart grid, and it is presented here clearly.

### 2.1 Communication system of smart grid

#### 2.1.1 SCADA

SCADA (Supervisory Control and Data Acquisition) functions as a controlling system and a network for communications within a smart electricity system [21]. Power system measurements can be gathered by the SCADA system, which possesses power system management and monitoring capabilities. The control center can estimate power grid state variables using this data, enhancing the electrical system's security and situational awareness. The power system's sensors monitor the instantaneous three-phase voltages, currents, and their phasors. Through a communication system, the control center receives these updates. To create precise directives for controlling the system using these estimates, the control center carries out a state estimation procedure. Real-time state estimation computes state variables based on field measurements made with meters. If the control center receives inaccurate readings due to cyberattacks, it will estimate the state incorrectly. Consequently, poor decisions will be made, potentially leading to the system's breakdown [22]. To achieve a high level of dependability and security, the information transfer system within the power grid should be made more resilient.

#### 2.1.2 AMI

Advanced metering infrastructure (AMI) systems can utilize either point-to-point or mesh communication architectures, allowing for local communication in close proximity or across longer distances [23,24]. AMI plays a fundamental role in the smart electrical system as one of its essential components. It is composed of advanced meters, sometimes referred to as smart meters, that monitor energy use, collaborate with one another to optimize energy consumption, and utilize data management systems to store and analyze metering and control data. AMI provides opportunities for better services, financial rewards, and the chance to address environmental problems [25]. As an essential component of the smart grid, AMI is tightly linked to people's daily lives [26]. AMI revolutionizes the electricity metering system by replacing outdated mechanical meters with advanced smart meters, enabling bidirectional communication between energy users and utility corporations. With the implementation of AMI, users can remotely read metering data, carry out fine-coarse demand management, and perform customized control [27].

#### 2.1.3 Smart meter

Smart meters are modern energy measurement devices utilized in households or businesses to gather data on electricity consumption from various devices. They analyze consumer energy usage, provide valuable information to utility companies or system operators, and enhance monitoring capabilities while streamlining billing processes. Smart meters monitor electrical parameters such as voltage and frequency, capturing real-time energy consumption data. They establish a connection between the residence or business and the smart grid, enabling bidirectional information and energy transfer. By facilitating two-way communication, smart meters establish a link between the meter and the central system, typically managed by the utility company or system operator [27]. From the end-user's standpoint, smart meters offer a variety of advantages, according to Ref. [27]. Users can predict their bills using the gathered data and reduce their energy usage to cut electricity costs. From the utility's vantage point, real-time pricing may be implemented using the data collected by smart meters. This enables them to set maximum power consumption caps and encourage users to consume less during high-load periods.

Undoubtedly, smart metering has received significant attention recently. Numerous countries, both inside and outside the EU, are already working on smart metering programs at the demonstration or larger scale. Smart meters are widely said to have several advantages, which are covered in depth in Ref. [28]. This claims that the electrical meter incorporates the “intelligence” of traditional meters. It can, among other things, measure the quantity of power used (or generated), remotely switch off the customer, and regulate the maximum amount of electricity use. Smart meters have benefits for many parties. As a result, distinct categories for energy users, grid operators, metering providers, suppliers, and governments are created. When prompted by market developments, smart meters can reduce or even stop energy usage. If every household and small to medium-sized business (SME) in a country could modify their energy consumption during high price or limited energy availability periods, it would enhance the reliability of the energy supply. Additionally, it would encourage energy market transactions, promote energy savings, increase awareness about energy usage, and improve overall energy efficiency.

**Fig. 4:**
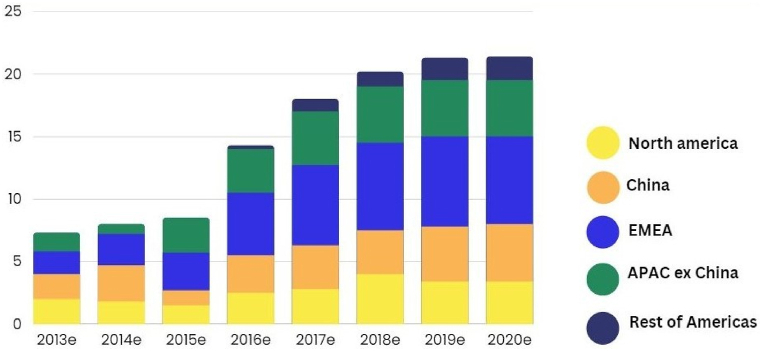
Global annual smart meter investment.

Global smart meter investments increased to 13 billion in 2018, with 800 million smart-meters installed worldwide before the year is through, as illustrated in Fig. 4. China has seen the most significant investments due to government targets, while Europe has mandated smart meter deployments with most member states aiming for installations in 80 percentages or more households by 2020. In the United States, More than half of all homes now have access to one of the 70 million smart-meters that have recently been implanted. The trend towards smart meter technology is expected to continue globally as countries strive to modernize their electrical grids and enhance energy efficiency [29].

### 2.2 Smart grid’s domains

**Fig. 5:**
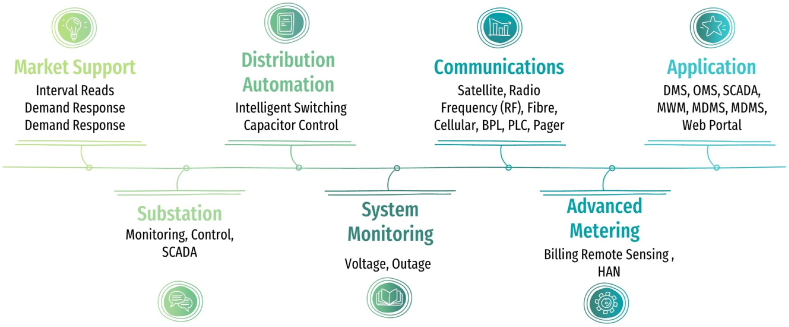
Seven domain in smart grid.

A modern electrical distribution system, referred to as a “smart grid,” incorporates advanced technologies such as sensors, communication networks, and analytics to enhance the efficiency, flexibility, and reliability of the grid. The smart grid comprises seven key components: Market Support, Demand Response (DR), Distribution Automation (DA), Communications, Advanced Metering Infrastructure (AMI), Electric Vehicles (EVs), Renewable Energy Integration (REI), Energy Storage, and cybersecurity. The implementation of smart meters, renewable energy sources, automation, electric vehicles, demand response, energy storage, and strong cybersecurity features are necessary for the development and success of a smart grid [30]. Fig. 5 presents the seven domains of the smart grid, offering a detailed overview of the essential components that form the foundation of this advanced electrical distribution system.

### 2.3 Embedded control module for smart grid

**Fig. 6:**
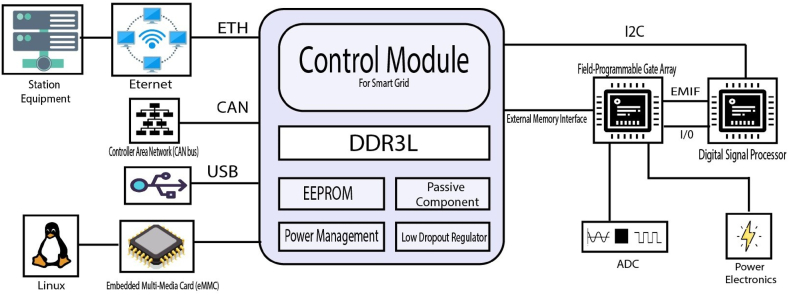
Embedded control module for smart grid.

Designing custom control systems that are significantly smaller, more dependable, and have better long-term support is now achievable for managing the traditional electric grid. This advancement is made possible by leveraging open-source initiatives and technologies like System-in-Package (SiP), which enables these systems to be as affordable and timely to deploy as conventional rack and stack solutions. Silicon Power Corporation's InnovaTM SCM21001 system-on-module (SoM) was developed as an embedded computing platform primarily for electric grid automation applications. The SCM21001 SoM integrates a real-time DSP subsystem with Octavo Systems' OSD3358 SiP, a Texas Instruments dual-core DSP, and an Intel Field Programmable Gate Array (FPGA). It also includes a Linux-based management controller. This single SoM optimizes DSP systems using conventional DSP and FPGA techniques, alongside management and monitoring software offering contemporary communication protocols and user interfaces. Designing a custom SoM solution instead of using the conventional Commercial Off-The-Shelf (COTS) approach allows for tight integration of application-specific components such as power, analog sensor conversion, and actuator drives. Its small size and ability to be conduction-cooled without fans enable full integration into high-reliability applications. Fig. 6 illustrates the modules for control mechanisms, with detailed descriptions provided. Workflow remained uninterrupted when transitioning from development platforms to the SOM for software development and system hardware design. In the initial application, exceptional results in solution size and performance were achieved, incorporating a bank of 32 simultaneously sampled 16-bit analog-to-digital converter channels directly under the SOM [31].

## 3 Vulnerabilities of smart grid

The introduction of enhancements and advanced capabilities into the smart grid network complicates the conventional electrical network and exposes it to various types of attacks. These issues can grant hackers access to the network infrastructure, compromise the security and integrity of transferred data, and disrupt service [32]. Critical vulnerabilities have been identified, as discussed in Refs. [33,34]. Physical security emerges as a primary vulnerability. Unlike conventional power systems, the smart grid network includes numerous components located outside the utility's premises, exposing them to physical trespassing risks. Additionally, the smart grid comprises intelligent components controlling electricity supply and demand, which could serve as potential entry points for cyber attacks. Managing and monitoring such a vast network of interconnected devices, known as the Internet of Things (IoT), poses significant challenges. Smart meters, for example, gather extensive data on consumer behavior, device usage patterns, and home occupancy, raising concerns about privacy and data security. The coexistence of power systems with IT infrastructure necessitates the use of outdated technologies, which may not integrate well with current system components, creating security vulnerabilities. Poorly coordinated team communication further exacerbates these vulnerabilities and can lead to critical decision-making lapses. Utilizing IP standards in smart grids offers compatibility advantages across all components. However, IP-based devices are susceptible to various network attacks such as IP spoofing, Denial of Service (DoS), and others.

## 4 Smart grid cyber physical security

**Fig. 7:**
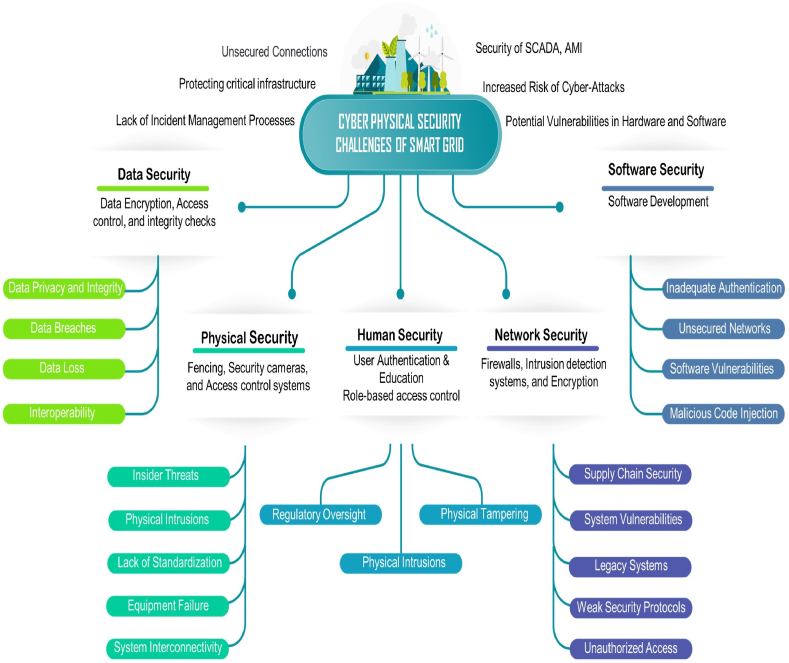
Cyber physical security of the smart grid.

Modern energy distribution systems, known as “smart grids,” integrate cutting-edge technologies such as au-tomation, communication networks, and sensors to enhance the efficiency, sustainability, and reliability of power supply. However, these systems are vulnerable to cyberattacks, which can significantly disrupt operations and cause substantial damage. Fig. 7 provides an overview of cyber-physical security, detailing total attack scenarios and security measures. Securing cyber-physical systems is crucial. This involves implementing various security measures, including data security, physical security, human security, network security, and software security. By adopting these measures, we can mitigate the risk of cyberattacks and protect our critical infrastructure, ensuring the reliability, safety, and resilience of our electrical systems [35].

Therefore, ensuring the security and reliable performance of the grid requires a tiered strategy for smart grid cyber-physical security.

## 5 Smart grid cyber attack classification

**Fig. 8:**
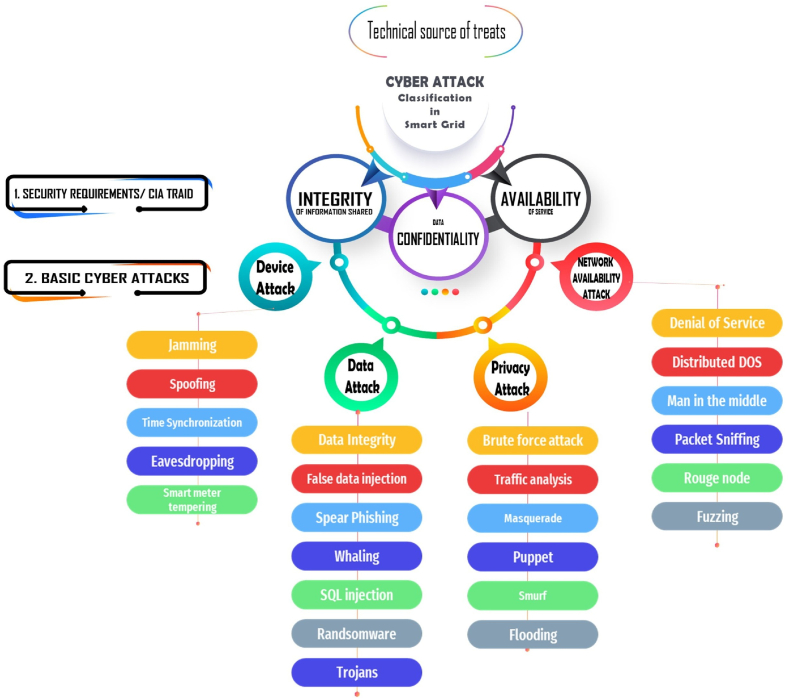
Cyber attack classification of smart grid.

In the study by Ref. [36], the author discusses the technical sources of threats to smart grid cyber-attack security, focusing on infrastructure, technical operations, and system data management security. According to CIA TRAID, cyber-attacks are classified into three types: Integrity of information shared, Data Confidentiality, and Availability of service [37]. These security objectives help categorize cyber-attacks into four main categories: Network Availability Attack, Privacy Attack, Device Attack, and Data Attack [38]. Specifically, the Network Availability Attack category includes threats to various network areas such as Wide Area Network (WAN), Home Area Network (HAN), and Neighborhood Area Network (NAN) [39]. In Ref. [40], the author discusses the five communication layers involved in network availability attacks: Transport layer, Application layer, MAC layer, Network layer, and Physical layer. Recent publications have focused on cyber-attacks targeting specific communication layers such as the network layer or physical layer. Three types of cyber-attacks—component-based, protocol-based, and topology-based—were explored in Ref. [32]. [41] classifies cyber-attacks into Operational Technology (OT), Advanced Metering Infrastructure (AMI), and Information Technology (IT) based attacks. Malicious hackers typically employ four methods—Scanning, Exploitation, Reconnaissance, and Sustain Access—to infiltrate and seize control over systems [42]. In this study, we have introduced a new classification of cyberattacks, as depicted in Fig. 8.

Cyberattacks targeting smart grids pose a significant threat to the stability, reliability, privacy, and security of electrical grids and consumers. Understanding the various types of cyberattacks is crucial for developing robust cybersecurity strategies. The Table 4 below summarizes the most prevalent types of cyberattacks on smart grids and their potential impacts.

**Table 4:** Cyber-attacks in smart grids.

Attack Name	Motive	First Reported (Year)	Location of the Demonstration	Malicious Threats
[4]DoS/DDoS	Attacks impede, delay, or harm in-formation exchange between Smart Grid nodes.	2015	Ukrainian power grid	Availability
[4]Malicious Software	Decreases or compromises avail- ability, integrity, or confidentiality of cyber infrastructure.	2014	Homeland Security's ICS-CERT	Integrity, Availability, Confidential-ity
[4]Identity Spoofing	Attackers pose as legitimate users without needing credentials.	2014	Dragonfly campaign	Integrity, Availability, Confidential- ity, Account-ability
[4]Password Pil- fering	Steals passwords compromising confidentiality.	2014, 2015	Dragonfly campaign, Ukrainian power company	Confidentiality
[4]Eavesdropping	Jeopardizes privacy of Smart Grid communications.	2011	University of South Carolina, University of California, Berke-ley	Confidentiality
[43] Intrusion	Unauthorized access compromising confidentiality and integrity.	2008	University of Cam-bridge	Confidentiality, Integrity
[44]Side-Channel Attacks	Exploits system construction to de-termine cryptographic keys.	2008	University of Califor-nia, Berkeley	Confidentiality
[45]Load-Redistribution Attacks	Attempts to cause overflow in smart grids.	2013	North Carolina State University, Carnegie Mellon University	Availability
[46]Data Injection Attacks	Manipulates real-time pricing via state estimator and measurement units.	2009	University of Illinois at Urbana- Champaign	Integrity
[47]Data Tamper-ing	Alters or removes data from smart grid systems.	2009	Control systems of a US power plant	Integrity
[48]Jamming	Interrupts communication signals within a network.	2010	University of Califor- nia, Berkeley, Uni- versity of Illinois at Urbana-Champaign	Availability
[49]Time Synchronization Attacks	Attacks timing information in smart grids.	2009	University of Illinois at Urbana- Champaign	Confidentiality, Integrity, Availability
[50]Smart Meter Tampering	Unauthorized manipulation of smart meters.	2012	FBI warning	Confidentiality, Integrity, Availability

Attack Name	Motive	First Reported (Year)	Location of the Demonstration	Malicious Threats
[51]Spear Phish-ing	Targets US utility company com-puter networks.	2014	Various US utility company networks	Confidentiality, Integrity
[52]Whaling At-tack	Targets executives for sensitive in-formation or access.	Not documented	Corporate and gov-ernment agencies	Confidentiality, Integrity
[53]SQL Injection Attacks	Injects malicious SQL code into vulnerable web applications.	Not documented	Organizations using databases	Integrity
[51]Ransomware Attacks	Disrupts critical systems and infras-tructure availability.	1989	Various industries	Availability
[54]Trojans	Disguises as legitimate software to gain unauthorized access.	1980	Stuxnet	Confidentiality, Integrity, Availability
[55]Brute Force Attack	Systematically guesses passwords or encryption keys.	Not documented	Critical infrastructure	Confidentiality
[56]Traffic Anal-ysis Attacks	Intercepts and analyzes network traffic for data.	Early 2000s	Smart grid adoption regions	Confidentiality
[57]Masquerade Attacks	Impersonates trusted users for unau-thorized access.	Early 2000s	Smart grid adoption regions	Integrity, Availability, Confidential- ity, Account- ability
[58]Puppet Attack	Attacks network layers to violate network availability.	Not documented	Smart meter systems	Availability
[48]Flooding At-tacks	Overwhelms systems with traffic or requests.	Early 2000s	Various industries	Availability
[59]Man-in-the-Middle Attack	Eavesdrops on or manipulates smart grid communications.	Not documented	Smart grid	Integrity, Confidential- ity
[40]Packet Sniff-ing	Intercepts and analyzes smart grid device communication.	Not documented	Smart grid	Confidentiality
[60]Rogue Node Attack	Adds unauthorized devices to dis-rupt smart grid communication.	Early 2000s	US, Europe, Asia	Confidentiality, Integrity, Availability
[61]Advanced Persistent Threats (APTs)	Long-term attacks targeting specific smart grid components.	2010	Stuxnet	Confidentiality, Integrity, Availability
[62]Insider Threats	Threats originating from within an organization.	2009	PG&E	Confidentiality, Integrity, Availability
[62]Social Engi-neering Attacks	Manipulates individuals for sensi-tive information or access.	2014	Ukrainian power grid	Confidentiality
[63]Teardrop At-tack	Causes errors in IP packet reassem-bly.	Late 1990s, early 2000s	Microsoft Windows systems	Availability
[64]Buffer Over-flow Attacks	Overflows data into adjacent mem-ory, compromising integrity and availability.	Known for decades	Smart grid	Integrity, Availability
[51]Popping the HMI Attack	Seizes control of Industrial Control Systems (ICS) for physical harm.	2014	German steel mill	Integrity

## 6 Cyber-attacks on smart grid

### 6.1 Device attack

Real-time grid status monitoring is made possible by the control center's advanced monitoring and control technologies. These technologies can promptly identify system flaws or disturbances and take corrective action. Additionally, they can assess grid utilization levels and adjust power levels as necessary to maintain stability and effectiveness. The smart grid (SG) consists of three primary components: Information Technology (IT), Operational Technology (OT), and Advanced Metering Infrastructure (AMI) [41].

OT describes the physical components and operational activities of industrial infrastructure controlled and monitored by hardware and software [65]. IT comprises storage servers, application servers, and servers for storing historical data. Smart meters and SG control centers can share data thanks to the AMI connection standard [66], safeguarding device-to-device communication using the ISO/IEC standard, as well as AMI, DCS, ICS, and SCADA as followed in Ref. [41].

**Fig. 9:**
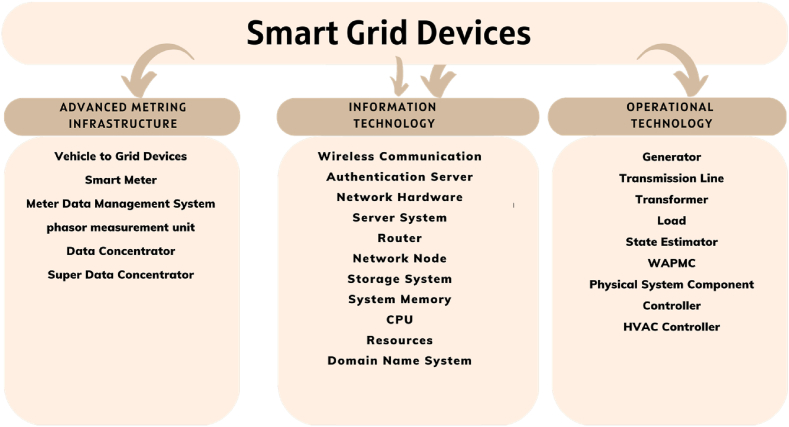
Smart grid devices.

The AMI framework facilitates communication among the SG control server, aggregators, and power consumers. AMI devices include smart meters, V2G devices, PMUs, MDMSs, DCs, and SDCs [67,68]. On the customer side, a smart meter is installed to track household electricity usage overall using HAN. Through NAN, data aggregators collect data from each customer and transfer it to the SG control server. The SG utilizes this information and data from the AMI network to maintain a steady power supply while considering demand from electricity consumers [41]. Fig. 9 provides an explanation of the smart grid devices.

### 6.2 Data attack

A deliberate introduction, modification, or removal of data or control commands within network traffic constitutes a “data attack,” aimed at causing the smart grid to make erroneous decisions or behave inappropriately. Manipulating a smart meter to reduce power costs often leads to a data breach. Similarly, a compromised Remote Terminal Unit (RTU) may detect an issue through a faulty circuit indicator (FCI) device but deliberately refrain from notifying the control center, prolonging the outage. Protecting data integrity and authenticity, along with developing effective intrusion detection techniques, are crucial defenses against such attacks [38].

### 6.3 Network availability attack

Network availability attacks, such as Denial of Service (DoS), aim to exhaust or overload the smart grid's com-munication and computing capabilities, causing delays or failures in data communications. For instance, adversaries may flood a control center with repetitive requests, inundating it with inaccurate information and preventing it from promptly responding to legitimate network traffic. In the context of the smart grid, where timely and accurate data are crucial for effective operation, even a brief delay can have severe consequences for homeland security and the national economy. Addressing network availability attacks requires effective and strategic mitigation measures [38].

### 6.4 Privacy attack

A privacy attack aims to obtain or infer private information about individuals by analyzing energy usage data. Smart meters in smart grids collect power usage data multiple times per hour to monitor grid status and improve operational efficiency. This detailed information can inadvertently reveal customers’ physical activities. For example, prolonged periods without power consumption from appliances like stoves and microwaves may indicate that a household is unoccupied, potentially facilitating targeted criminal activities such as burglaries. Safeguarding such sensitive data from unauthorized access is paramount [38].

## 7 Cyber-attack detection

### 7.1 Device attack detection

#### 7.1.1 Machine learning technique- Support Vector Machine (SVM)

Customizing a machine learning technique like Support Vector Machines (SVM) enables the identification of potential attacks within devices before they occur. Additionally, TFPG, a mechanism for analyzing attack paths, is employed to discover these paths. During experiments, it was found that the SVM classifier requires shorter training times compared to a Neural Network (NN) classifier, while effectively and accurately detecting attacks on smart meters. A real-time Fault Detection and Identification (FDI) model is provided by a maximum likelihood estimator based on observations of parameters such as power flow, voltage magnitudes, or phase angles. Through extensive training using a diverse set of both normal and abnormal IDS events, the SVM learning model can effectively distinguish between abnormal and normal occurrences in FDI assaults [69].

### 7.2 Network based detection

**Fig. 10:**
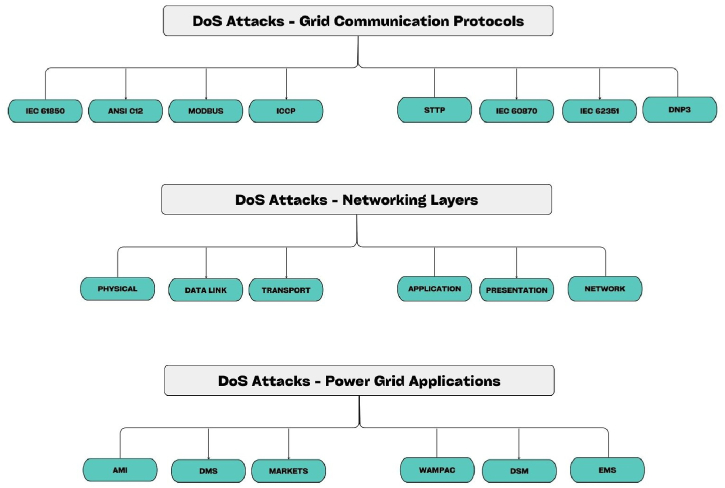
DoS attack in SG in terms of (a) communication protocol, (b) Networking layer, (C) power grid application [70].

Denial of service (DoS) is an attack type where attackers try to unavailable data and information for the desired users by attacking the server of the smart grid. Fig. 10 informs us that attacker Attacks servers of the smart grid and finally managed some server to be compromised so that they can control the client program. Here the whole process is based on a central network and around this network there are server, computer links, attackers are being placed so this system is like an internet where the same architecture is made [70].

This type of attack can be in the layer of the smart grid. DoS mainly try to collapse all of the communication layer first and then it tries to make physical, data link layer, transportation layer etc in their control. DoS attacks all of the segment of the SG [71]. According to Ref. [71], it also affects in the power grid applications and the smart metering services which is very much emerging. The layer of the smart grid can be susceptible to two distinct types of attacks, jamming and tempering. Jamming mainly works under basic communication like sender and receiver and tempering [6]. From.

[70] we find a high-level categorization of DoS and finally various source were discussed and thus we got the idea how it attacks in different IP and collapse them. In the Fig.10 [70] we explored DoS attack on network protocol, communication layer and important SG application. There can be multiple method of intrusion detection. Here in Ref. [8], by using genetic algorithm a model is proposed to mitigate DoS.

### 7.3 Data attack detection

A data attack aims to maliciously introduce, delete, or modify data or control commands within network traffic to induce incorrect judgments or actions in the smart grid (SG). For instance, attackers may manipulate smart meters to reduce electricity bills [38]. Among these, False Data Injection Attacks (FDIAs) pose a significant threat by using false data to deceive smart meters in power grids and manipulate measurements [72]. Malware injection, such as viruses or worms, is another common type that compromises system integrity. FDIAs alter data without modifying the system's code and can potentially manipulate device inputs at the physical layer to generate false or inaccurate results [73]. By manipulating sensor measurements within SG, FDIAs can target all levels of SG systems while bypassing traditional defenses [73]. This section focuses on FDIAs, their mitigation strategies, and related data attack tactics.

#### 7.3.1 Machine and deep learning based

Machine learning-based techniques for detecting data anomalies have become widely adopted in cyber-attack detection. According to Cui et al. [73], these techniques primarily involve detecting abnormal energy consumption data. They can be categorized into two main groups: supervised and unsupervised machine learning algorithms. Support Vector Machine (SVM) techniques are frequently utilized due to their advantages over traditional classifiers, particularly in identifying energy theft. Recent advancements include hybrid SVM-based algorithms and discussions on deep learning techniques. The evolving landscape of smart grids, influenced by renewable energy sources and topology changes, presents challenges for cybersecurity defenses against malicious cyber-attacks. Mohammadpourfard et al. [74] proposed a detection technique robust to system setting and topology changes. Niu and X [75] introduced a framework based on deep learning to detect measurement irregularities caused by False Data Injection (FDI) attacks, leveraging recurrent and convolutional neural networks. Yan et al. [76] conducted a comparative study on supervised learning classifiers for detecting counterfeit data in smart grids, highlighting their effectiveness in binary classification tasks. They emphasized the role of machine learning detectors in identifying and mitigating FDI attacks, which can disrupt operations by providing false measurements. Sengan et al. [77] introduced True Data Integrity using an Agent-Based Model to quantify attack exposure, focusing on decentralized data integrity security within systems. Alamin et al. [78] proposed a hybrid model combining deep learning and machine learning methods to enhance detection rates while ensuring reliability.

#### 7.3.2 Using graph signal

**Fig. 11:**
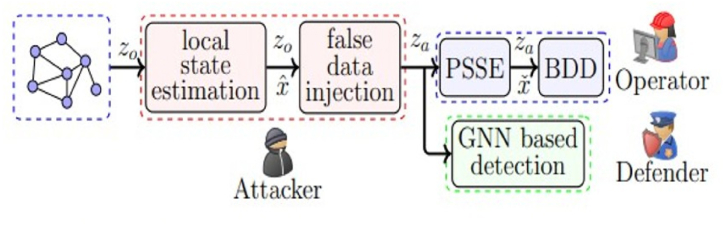
Architectural overview and signal flow graph proposed in Ref. [72].

Conventional residual-based techniques for detecting bad data are limited in their ability to identify risks arising from the injection of fake data (FDI). The method proposed in Ref. [79] filters the predicted grid state, computes high-frequency elements using the Fourier transform of the graph, and aims to detect Alon's FDI attacks, which aim to disrupt Power System State Estimation (PSSE). This process is integral to the SCADA system of an electric grid's control center. Fig. 11 outlines the steps and provides an architectural overview of the signal workflow.

According to Ref. [79], a model of the power system is represented as an undirected graph. They also present an evaluation of FDI attack detection as a hypothesis testing problem after introducing the AC power flow model.

In a related approach to uncovering anomalies in power usage [80], discusses a graph-based anomaly detection method. They apply this technique to analyze real-world data, specifically focusing on electrical usage patterns in smart grids. The method, known as GBAD, utilizes vertices to represent smart appliances and edges to denote utilization between different components within a home. The authors report high precision, recall, and accuracy in identifying anomalies. However, it is noted that GBAD may be vulnerable when attackers target multiple packets from the same smart grid device.

Another approach to identify FDIA is presented in Ref. [72], which describes a general, localized, and stealthy attack generation method, as shown in Fig. 11. The paper also provides publicly accessible datasets for researchers to develop and test their algorithms. Leveraging spatial correlations of measurements and integrating physical interconnections within AC power grids [72], proposes a scalable, real-time Graph Neural Network (GNN)-based FDIA detector that combines design and data-driven strategies effectively.

The concept of graph signal processing models smart grids as graphs, where nodes represent different grid components and edges represent interconnections. Each component is associated with a signal such as voltage or current readings, enabling comprehensive analysis of the grid's behavior.

#### 7.3.3 Using tree-based algorithm

Hackers with malicious intent can manipulate SCADA readings by injecting biased values into sensor-collected measurements, aiming to deceive bad-data detectors during state estimation. This manipulation could lead to incorrect control decisions that compromise the smart grid's security, causing financial losses, network disruptions, or both. In older systems, a bad data detector (BDD) evaluates the accuracy of sensor-acquired measurement data. Recent findings highlight the stealthy cyber-attack (SCA) described in Ref. [81], which can evade traditional BDDs. A skilled hacker can exploit this attack vector to manipulate sensor data, introducing biased values. In addressing such challenges [82], propose a novel method using an algorithm based on extremely random trees and Principal Component Analysis (PCA). This approach aims to detect Side-Channel Attacks (SCA) within Smart Grid (SG) networks. To manage the computational complexity of large-scale power systems, the authors employ Kernel PCA (KPCA) to reduce dimensionality. The processed data is then fed into the Extra-Trees algorithm, known for its speed and effectiveness in detecting SCA.

#### 7.3.4 Using energy consumption forecasting

The algorithm described operates on a data-driven approach, eliminating the need for specific model or system parameters. This contrasts with model-based detection algorithms, which rely heavily on precise system characteristics that, if unclear or inaccurate, can significantly impact their performance [83]. In Ref. [84], a two-step anomaly detection engine is proposed, leveraging a CNN-LSTM Autoencoder named FDI (False Data Injection). This approach not only identifies intrusions but also evaluates deviations of field readings from expected values. Experimental results demonstrate that the CNN-LSTM Autoencoder achieves superior accuracy in predicting datasets, underscoring its efficacy in anomaly detection applications. Furthermore, as highlighted in Ref. [85], the successful detection of abnormal activities in anomaly detection approaches hinges on accurate predictions derived from real-time data. In the domain of intrusion detection systems (IDS) [86], introduces SafetyMed, a novel system that integrates LSTM networks with CNNs to safeguard against intrusions originating from grid and data sources. SafetyMed achieves an impressive average accuracy of 97.63 % and average precision, showcasing its robust performance in intrusion detection.

### 7.4 Privacy attack detection

In the context of privacy attacks targeting electricity usage data, smart meters deployed in smart grids collect detailed information about power consumption multiple times per hour, potentially revealing sensitive information such as occupants’ daily routines. Detecting data leakage from usage patterns is crucial in this scenario. Traditional occupancy detection systems typically rely on specialized sensors like cameras, magnetic reed switches, or passive infrared (PIR) sensors. Recent research, however, explores the use of digital power meters, widely installed in millions of homes worldwide, as effective occupancy sensors [87]. An eight-month study in five households gathered ground truth occupancy data using an Android app developed for this purpose. Additionally, data from PIR sensors and individual appliance electricity consumption provided indirect validation for their findings. Furthermore, flexible sensor devices distributed widely in ordinary residences can contribute to cost reduction and system reliability in occupancy detection [88]. For example, smartphones can be leveraged to determine residential occupancy. Addressing privacy concerns [89], introduces a novel intrusion detection tool using correlation coefficient EM clustering techniques on SCADA data. This method effectively identifies less sensitive information from SCADA datasets and applies EM clustering to detect anomalous activities, demonstrating superior performance compared to other methodologies in identifying SCADA attacks. Energy theft remains a significant challenge in power infrastructure, evolving alongside advancements in smart grid technology like Advanced Metering Infrastructure (AMI). Researchers have developed threat models based on attack trees to address energy theft within AMI systems [90]. Various detection schemes categorized by their core principles are proposed to combat this issue. Common methods of energy theft include complete meter bypassing [91] and meter tampering [92]. Research such as [93] enhances predictive models to detect technical losses in distribution networks, considering factors like temperature effects on circuit resistances. These models are evaluated across different levels of power theft to determine their effectiveness in reliably detecting instances of theft.

## 8 Cyber-attack defense

### 8.1 Device Attack defence

#### 8.1.1 Dynamic bayesian honeypot game model based

An adversary can be misled by a honeypot deployed on a network, tricking them into believing a simulated system is an actual power grid infrastructure. Within a virtualized environment, this honeypot gathers intelligence on the attacker's behavior, functioning as a countermeasure that forces the attacker to expend resources attempting to breach the honeypot ecosystem. However, most honeypot systems traditionally employ static defense tactics, which pose challenges in dealing with dynamic threats. Moreover, attackers can easily circumvent a honeypot by detecting the artificial environment, such as through anti-honeypot techniques like those observed in Singapore. Alternatively, a dynamic Bayesian honeypot game model serves as a deterrent against attackers aiming to execute dynamic Distributed Denial of Service (DDoS) attacks within the Advanced Metering Infrastructure (AMI) [94]. This model leverages Bayesian Nash equilibriums to optimize defensive strategies, enhancing attack detection accuracy and minimizing energy consumption for defense purposes. Despite potential anti-honeypot methods employed by attackers, the dynamic honeypot defense system effectively mitigates these evasion tactics, thus enhancing predictive capabilities for optimal AMI network defense strategies.

#### 8.1.2 Game theory based on the tree-structured analysis

The application of a game theory framework, utilizing tree-structured analysis, provides significant advantages in efficiently allocating resources within the smart grid (SG) and formulating appropriate defense scenarios. This framework focuses on evaluating the effectiveness of specific attack strategies. It utilizes the tree-structured model to illustrate various attack paths, demonstrating multiple attack methods [95].

#### 8.1.3 Asymmetric hash-based encryption schemes

SG is susceptible to cyberattacks targeting Electric Vehicle (EV) charging stations since the infrastructure for charging electric vehicles (EVs) is built on the SG [69]. Additionally, because EVs are mobile and exchange sensitive data with the charging stations, Protecting the infrastructure from cyberattacks in the SG is complex compared to securing other systems. In light of these security considerations, the SG-based EV charging infrastructure should be created. Potential solutions for ensuring secure communication in Vehicle-to-Grid (V2G) systems can include asymmetric hash-based encryption algorithms and bidirectional authentication processes [96]. For EV charging system security objectives and requirements, The NISTIR 7628 framework is using as a security framework [97].

#### 8.1.4 Encryption algorithms based

Smart meter transmissions can be secured through encryption methods, where the encryption key plays a crucial role. Proper management of encryption keys within the Advanced Metering Infrastructure (AMI) network is essential, particularly when dealing with numerous meters between nodes. In their article, the authors propose a practical paradigm for key management to ensure secure smart meter communication. They also introduce an effective technique for generating new keys and modifying existing ones, addressing both time and space complexities.

### 8.2 Data attack defence

Data integrity attacks, particularly false data injection (FDI) and bad data injection (BDD) attacks, are among the most worrisome types of data assaults. We talked about detecting methods in the previous part, and now we're going to suggest some defense methods that can lessen data attacks.

#### 8.2.1 Using concept drift

In the realm of machine learning, ‘concept drift’ refers to sudden changes in the underlying distribution of past data over time, indicating abrupt shifts in the data characteristics [98]. These shifts can significantly impact the effectiveness of models trained on historical data, requiring adaptive strategies to maintain accuracy and relevance as new data arrives. The smart grid faces potential threats from False Data Injection (FDI) attacks, which can compromise its management and operation. Addressing this challenge, a paradigm proposed in Ref. [99] advocates for resilience in essential algorithms. Instead of relying solely on historical data as a baseline when updating training sets, the approach recommends sampling from critical concept sets that reflect substantial changes in data dispersion from the baseline concept. By focusing on critical concept sets, this strategy aims to improve the robustness of machine learning algorithms against concept drift induced by FDI attacks in smart grid environments. This proactive approach ensures that models can adapt effectively to evolving data patterns, thereby enhancing the security and reliability of smart grid operations.

#### 8.2.2 Multi-agent based system

In [100], a novel approach leveraging multi-agent design is explored to enhance Self-Adaptive Intrusion Prevention (SIP) systems, focusing on context awareness and self-adaptiveness. This decentralized setup emphasizes data-driven anomaly detection within cyber-physical systems (CPS), particularly in power grids. The study successfully develops a comprehensive taxonomy of operating states, which transforms the anomaly detection problem into a multi-class classification task.

The Multi-Agent System (MAS)-based rule-based intrusion detection approach proposed in Ref. [100] aims to enhance the security protocols of cyber-physical energy systems. This approach utilizes a multi-agent strategy to facilitate secure data transfer between agents, emphasizing state-aware protocols as outlined in Ref. [101]. This protocol employs a supervised multi-class classification algorithm to accurately identify anomalies within CPS operating states.

Overall, the integration of multi-agent systems and advanced classification algorithms represents a significant advancement in enhancing the security and resilience of cyber-physical energy systems, offering robust protection against evolving cyber threats.

#### 8.2.3 Using adaptive CUSUM test

In addressing the challenge of defending against fake data injection attacks in smart grid networks, a non-Bayesian framework known as the CUSUM (Cumulative Sum) test has emerged as a promising solution. Unlike Bayesian methods, the CUSUM test detects changes in distributions from known to unknown at random intervals, making it adaptable to varying temporal distributions and unknown patterns. An adaptive CUSUM algorithm has been proposed specifically to mitigate fake data injection assaults within smart grid networks. This approach involves two phases integrated into the smart grid state estimation system, as illustrated in the diagram referenced in Ref. [102]. The adaptive CUSUM method aims to maintain high detection accuracy while minimizing detection delays. According to the findings in Ref. [102], the adaptive CUSUM technique has demonstrated excellent performance in achieving targeted detection accuracy levels. It is noted for its simplicity, effectiveness in accurate detection, and ability to maintain a low average run length, which is critical for timely response to potential threats in smart grid environments. This approach highlights the importance of robust detection mechanisms tailored to the unique challenges of smart grid cybersecurity, ensuring the reliability and integrity of grid operations amidst evolving cyber threats.

#### 8.2.4 Adaptive markov stratigy

In [103], there is a growing trend towards adopting game-theoretic frameworks to analyze interactions between attackers and system defenders, and to develop defensive strategies using game-theoretic techniques. This approach provides a theoretical basis for understanding how adversaries behave and how defenders can optimize their responses. An innovative adaptive method known as AMS (Adaptive Multi-Stage) is introduced in Ref. [104]. AMS is designed to dy-namically adjust defensive strategies based on evolving threats and system conditions. It has been theoretically proven to be logical and convergent, indicating its effectiveness in practical applications. Moreover, extensive experimental studies conducted in Ref. [104] demonstrated AMS's superiority over traditional Nash Equilibrium (NE) techniques in combating a range of cyberattacks on power distribution systems. Specifically, AMS showed improved performance in scenarios involving attacks such as fake data injection, highlighting its practical efficacy and robustness under various testbed settings. These advancements underscore the importance of adaptive and game-theoretic approaches in enhancing cybersecurity for power distribution systems, offering insights into effective defensive strategies against evolving cyber threats.

### 8.3 Network based defence

To address the challenge of mitigating Denial of Service (DoS) attacks in smart grids, a multifaceted approach integrating various techniques is essential, as highlighted in Ref. [105]. Specifically, no single solution exists for DoS mitigation, necessitating the integration of multiple techniques. From Ref. [106], a non-technical method for IoT security provides insights applicable to DoS mitigation in smart grids. This approach focuses on preventing unauthorized access, emphasizing its non-technical nature within the smart grid context. It includes elements of risk assessment and risk analysis crucial for enhancing security posture [107]. Filtering emerges as an effective technique, particularly when attackers and sources are in close proximity, as discussed in Ref. [70]. Distributed Packet Filtering (DPF), as outlined in Ref. [108], involves packet forwarding/discard and filter table updates at different time scales, ensuring near-line-speed performance. Implementing route-based DPF can effectively prevent fraudulent IP flows from going undetected. Intrusion Detection Systems (IDS) play a pivotal role in monitoring entire traffic streams, including headers and payloads. Utilizing genetic algorithms, as discussed in Ref. [109], enhances the capability to detect and mitigate intrusions effectively within smart grid environments.

**Fig. 12:**
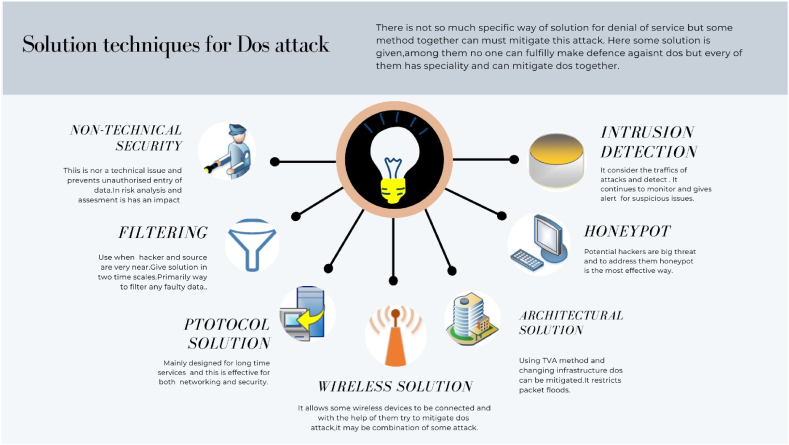
Solution techniques for DoS attacks in smart grid.

Integrating these techniques forms a robust defense mechanism against DoS attacks in smart grids, combining technical and non-technical approaches to bolster cybersecurity defenses comprehensively. Addressing Denial of Service (DoS) attacks in smart grids requires a multifaceted approach that incorporates various techniques to bolster cybersecurity defenses. Rate limiting is one such technique aimed at reducing network traffic, which can aid in detecting and mitigating DoS attacks. This can be implemented at perimeter devices like reverse firewalls and logically on server machines [110]. Encryption, while essential for securing data, can itself become a target for DoS attacks if the process of verifying packet validity consumes significant resources. Attackers can exploit this vulnerability using counterfeit packets to overload systems [70]. Countermeasures discussed in Ref. [111] highlight schemes resistant to cryptographic DoS attacks, emphasizing the importance of robust security protocols. Future concerns include protocol-level attacks on smart grid (SG) infrastructures, similar to historical oversights in IP and UDP/TCP protocols [112]. To ensure long-term security, SG protocols must prioritize evolvability, allowing for updates and improvements over extended device lifespans [113]. Infrastructure changes, such as Traffic Validation Architecture (TVA), play a crucial role in mitigating DoS impacts by strictly limiting the effects of packet floods [114]. Honeypot systems are also employed to deceive attackers and divert them from the main system, thereby enhancing overall security [115]. Innovations like selectively substituting genuine devices with honeypots represent novel approaches to balancing connectivity and security in smart grids [116]. Wireless communication presents both opportunities and challenges in SG security. Effective jamming detection systems are essential due to stringent latency requirements, ensuring reliable message delivery in the face of jamming attacks [116]. Fig. 12 illustrates various solution techniques for DoS attacks, emphasizing the importance of intrusion detection systems, firewalls, encryption, and a layered security approach to safeguard smart grids from evolving cyber threats. In conclusion, protecting smart grids from cyber threats demands continuous vigilance and the adoption of advanced defense mechanisms. By integrating multiple techniques and staying ahead of emerging threats, smart grid operators can maintain robust cybersecurity postures essential for reliable and secure energy distribution.

### 8.4 Privacy attack defense

Smart meters are increasingly replacing traditional electromechanical ones because of their numerous advantages. They can adjust load for demand response, develop relationships between utility services and end users, and save energy. However, while smart meters offer fine-grained usage data, this also creates new vulnerabilities for both customers and companies. One such instance of privacy violation is occupancy detection. Given the close relationship between occupancy and power use, detecting a home's occupancy is straightforward using time-of-use data. An effective countermeasure, the AMLODA model, is presented in Ref. [117] to enhance user privacy. The goal of the suggested approach is to optimize the schedule for rescheduling consumption data from smart meters, thereby enhancing privacy protection. Additionally, it caters to users' wishes to provide various essential levels of anonymity. The system maintains payment accuracy while employing the suggested customer-oriented approach, which offers very high levels of privacy. This makes the model's adaptation practical because no extra hardware devices or trusted third parties are required.

Smart meters unintentionally reveal private data about a home's occupancy, which may be easily found because it strongly corresponds with fundamental statistical measures like the mean, variance, and range of power. Recent research [87,118] reveals that occupancy significantly correlates with some parameters, and due to the presence of fundamental statistical parameters such as mean, variance, and range of power data, attackers can exploit vulnerabilities to extract occupancy information from smart meter data with relative ease. In line with [119], the method known as Combined Heat and Power (CHPr) eliminates occupancy detection by leveraging the large elastic heating loads, particularly electric water heaters, already installed in many households. CHPr utilizes thermal energy storage as a means to conceal occupancy, as employing chemical energy storage, such as batteries, would require a costly level of energy storage capacity. The CHPr method incorporates activity- and occupancy-aware optimizations, artificial power signature injection, and partial demand flattening to reduce its energy requirements. It's crucial to note that CHPr doesn't waste energy or drive up electricity prices. The CHPr technique achieves occupancy concealment by rescheduling the energy consumption of a water heater. Additionally, the CHPr approach has been subjected to advanced occupancy detection attacks utilizing techniques such as k-NN clustering, Hidden Markov Models (HMMs), Support Vector Machines (SVMs), and thresholding [119]. According to Ref. [120], the actual demands for sensitive meter data in the electrical supply industry may not be as significant as anticipated. In the context of the smart grid, the utilization of smart meter data gathered from individual households is predominantly important for the operation and planning of the distribution system. However, privacy concerns need to be addressed by improving the way homes are classified within network designs to preserve privacy.

In the examination of cybersecurity measures within smart grid infrastructures, a comprehensive understanding of detection and defense methods against cyber attacks is crucial. Table 5 presents an overview of the detection and defense methods deployed to mitigate various types of cyber threats encountered in smart grids. The table outlines the specific detection techniques and corresponding defense mechanisms employed to safeguard against attacks such as Denial of Service (DoS/DDoS), malicious software, identity spoofing, and more. By analyzing the strategies outlined in this table, researchers and practitioners can gain insights into the multifaceted nature of cybersecurity in smart grids and devise robust defense strategies to mitigate potential threats.

**Table 5:** Detection and defense methods of cyber attacks in smart grid.

Type of Attack	Detection Methods	Defense Methods
[121] DoS/DDoS	Network traffic analysis, anomaly detection	Network traffic filtering, traffic throttling
[51] Malicious Software	Anti-virus software, intrusion de-tection systems	Software patches and updates, fire-walls, network segmentation
[122] Identity Spoofing	Multi-factor authentication, IP ad-dress filtering	Strong passwords, digital certifi-cates
[123] Password Pilfering	Password managers, multi-factor authentication	Strong passwords, encryption, reg-ular password changes
[55] Eavesdropping	Encryption, secure communication protocols	Network segmentation, firewalls
Intrusion	Intrusion detection systems, log monitoring	Intrusion detection systems, log monitoring
[124] Side-Channel Attacks	Cryptography, secure hardware de-sign	Physical security measures, secure communication protocols
[125] Load-Redistribution Attacks	Anomaly detection, monitoring of power system parameters	Enhanced power system control mechanisms
[126] Data Injection Attacks	Anomaly detection, integrity checks	Authentication, encryption, intru-sion detection systems
[48] Jamming	Signal analysis, power system mon-itoring	Enhanced power system control mechanisms, frequency hopping
[127] Time Synchronization At-tacks	Secure time synchronization proto-cols, encryption	Network segmentation, secure hard-ware design
[128] Smart Meter Tampering	Physical security measures, tamper-proof seals	Regular inspections, secure com-munication protocols
[129] Data Tampering Attacks	Data integrity checks, anomaly de-tection	Authentication, encryption, intru-sion detection systems
[130] Spear Phishing	Employee training, spam filters	Multi-factor authentication, network segmentation
[51] Whaling Attacks	Employee training, access controls	Multi-factor authentication, regular password changes
[131] SQL Injection Attacks	Input validation, parameterized queries	Regular software updates, secure coding practices
[40] Ransomware Attacks	Anti-virus software, intrusion de-tection systems	Regular software updates, data backups
[132] Trojans	Anti-virus software, intrusion de-tection systems	Regular software updates, network segmentation
[133] Brute Force Attacks	Account lockout policies, multi-factor authentication	Strong passwords, account monitor-ing
[51] Traffic Analysis Attacks	Anomaly detection, network traffic analysis	Encryption, network segmentation
[134] Masquerade Attacks	Multi-factor authentication, access controls	Regular password changes, intru-sion detection systems

Type of Attack	Detection Methods	Defense Methods
[135] Puppet Attack	Network traffic analysis, anomaly detection	Network segmentation, firewalls
[136] Smurf Attack	Network traffic analysis, anomaly detection	Network traffic analysis, anomaly detection
[48] Flooding Attacks	Network traffic analysis, anomaly detection	Network traffic filtering, traffic throttling
[137] Man-in-the-Middle Attacks	Encryption, secure communication protocols	Digital certificates, secure hardware design
[40] Packet Sniffing	Encryption, secure communication protocols	Network segmentation, intrusion detection systems
[60] Rogue Node Attack	Authentication, access controls	Secure communication protocols, regular software updates
[138] Advanced Persistent Threats (APTs)	Anomaly detection, network traffic analysis	Network segmentation, intrusion detection systems
[139] Insider Threats	Employee training, access controls	Employee monitoring, network seg-mentation
[140] Social Engineering Attacks	Employee training, access controls	Multi-factor authentication, spam filters
[141] Teardrop Attacks	Network traffic analysis, anomaly detection	Network traffic filtering, traffic throttling
[142] Buffer Overflow Attacks	Code reviews, input validation	Software patches and updates, se-cure coding practices

In the next section, we present Table 6 which outline key details including Author, Dataset, Method, Main Contribution, and Result. These tables serve as a comprehensive reference point for the methodologies and findings discussed in the subsequent analysis. Each entry encapsulates the essence of the respective study, providing valuable insights into the approaches employed, the datasets utilized, the main contributions made by the authors, and the resulting outcomes. This structured presentation aids in the synthesis and evaluation of the various research endeavors within the scope of our investigation, offering a holistic perspective on the advancements and discoveries in the field.

**Table 6:** Cyber attacks in smart grids: Author, dataset, detection method, Defence method, main contribution, result.

Author	Cyber Attack	Dataset	Detection Method	Defence Method	Main Contribution	Result
rao2024novel [143]	DoS/DDoS	NSL-KDD or real-time data from Wireshark or Hping3	LSVM, MLP, LSTM models	Traffic filtering, Fire-wall rules, Flask Rate Limiter, Honeypot	Detection and mitigation techniques, Enhanced security, Future research directions	LSVM: 96.69 %, LSTM: 87.64 %, MLP: 97.80 % Mitigation: Effective
eder2017cyber [140]	Malicious software	CICIDS 2017, NSL-KDD, UNSW-NB15, DARPA IDS, CTU-13	Anomaly Detection, Extended Firewall Use	Data Backup Strategies	Analysis of existing malware, Prediction of future threats, Defense strategies	Comprehensive malware analysis, Defense enhancements
kosmanos2020novel [144]	Spoofing attacks	Simulated data using SUMO, OMNET++/VEINS, GEMV tool	k-Nearest Neighbor, Position Verification	Not specified	Probabilistic IDS using Machine Learning, Novel spoofing detection metric	IDS achieved 91.3 % ac-curacy
zhang2024timing [145], wang2024secure [146]	Side-Channel Attack	Dragon_Pi IoT intru-sion detection dataset	AI-based intrusion detection models (code detection, behavior detection)	Secure scan architecture (dynamic key, CC-Hunter, Cyclone, PerSpectron, EVAX, SPOILER-ALERT)	Introducing Dragon_Pi dataset and Dragon_Slice for anomaly detection	AUC: 0.764 (without post-processing), AUC: 0.89 (with MAF on MSE length 17)
Pinceti2022 [147]	Load Redistribution At-tacks	Normative and anoma-lous load data	Nearest-neighbor-based detector	Localizing and assess-ing attack likelihood on system loads	Detection and localiza-tion on large-scale sys- tems	Average log-loss: 0.340, 0.489, 0.608
Niu2015 [148]	Jamming Attack	Real-time data from backbone communication network	Not specified	Online optimization and linear programming approach	Anti-jamming communication technologies (DSSS, FHSS)	Evaluated based on av-erage throughput and similarity of SU knowl- edge
iqbal2024cybersecurity [149]	Smart Meter Tamper-ing	Not mentioned	Intrusion Detection and Prevention Systems	Encryption, Authentication, Access Control, Security Audits	Cybersecurity in smart metering systems	Not mentioned
yan2024game [150], chukwue- meka2024detection [151]	False Data Injection	IEEE datasets, RTDS-based experiments	Graph Autoencoder Graph Convolutional, Network, Deep-Q-learning	Game theory-based re-source allocation	ML/DL techniques for detection, RTDS-based defense experiments	Detection: 84 %–86.1 % accuracy, Enhanced de-fense strategies
nahmias2024prompted [152]	Spear Phishing Attacks	Automated proprietary system for reconnais-sance and email cre-ation	Prompted Contextual Vectors	Not mentioned	Document vectorization leveraging LLMs' reasoning capabilities	F1 Score: 91 %
Zaim2019 [153]	Masquerade Attacks	SEA, Greenberg, PU, WUIL datasets	BDT, SVM, ANN, LDSVM, DF, DJ	–	Masquerade detection	BDT: 0.7818, SVM: 0.8096, ANN: 0.7561, LDSVM: 0.8423, DF: 0.8895, DJ: 0.9084
Patrick Wlazlo2021 [154], Bhushan2017 [155]	Man-in-the-Middle (MITM) Attacks	Inverters to cloud server data transit	Blockchain-based MITM detection	Router and Host-based solutions	Detection of advanced MITM attacks in PV systems	Security status provi-sion for PV system as- sets
moradi2024petri [156],	Time Synchronization	C/No measurements	Petri net model	Cross-layer detection	Importance of formal	Detection: 0.68 %
Zhang2013 [155]	Attacks/Pulse Delay Attackad	from GPS receivers		mechanism	modeling tools (CPN) for network security	overhead due to PTP algorithm, Defense: faster suspicious level increase under TSA

## 9 Self- healing of smart grid

Electricity users can actively participate thanks to the self-healing capabilities of smart grids. Smart grid technologies are self-healing systems that reduce the burden and strive to provide all users with sustainable, dependable, and high-quality power and can quickly identify solutions to problems in an existing system [157]. In this section, we have assessed the network's capacity for self-healing in scenarios involving cyberattacks, microgrids, transient states, and transmission.

**Fig. 13:**
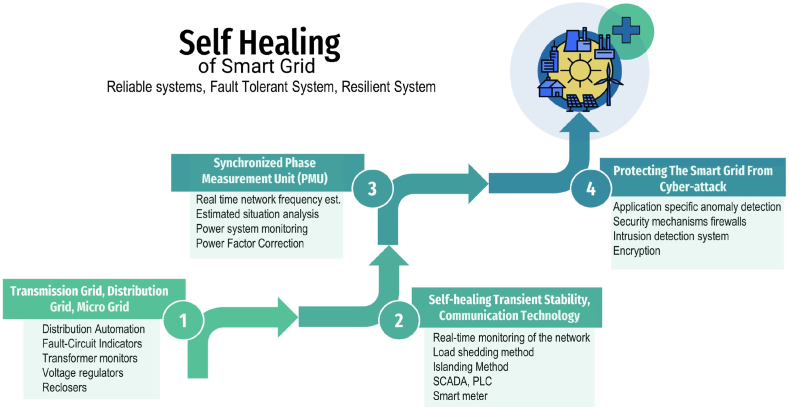
Self-Healing of smart grid.

Excluding production-related grids such as Transmission, Distribution, and Micro, the smart grid's power system comprises three basic grids. A shorter self-healing time in the network leads to reduced energy reserves and a limited timeframe for system regeneration. Wide area monitoring, protection, and control (WAMPAC) utilize the intuitive algorithm-based design of the integer linear programming (ILP) model to safeguard against cyberattacks, incorporating cryptography, access control, and firewalls. This approach is crucial in protecting the smart grid from cyberattacks by facilitating self-healing mechanisms, such as the reconnection of Phasor Measurement Units (PMUs) and the restoration of system observability [158]. In Fig. 13, we can observe the self-healing process of a smart grid, where it undergoes several steps.

## 10 Prospective direction

**Fig. 14:**
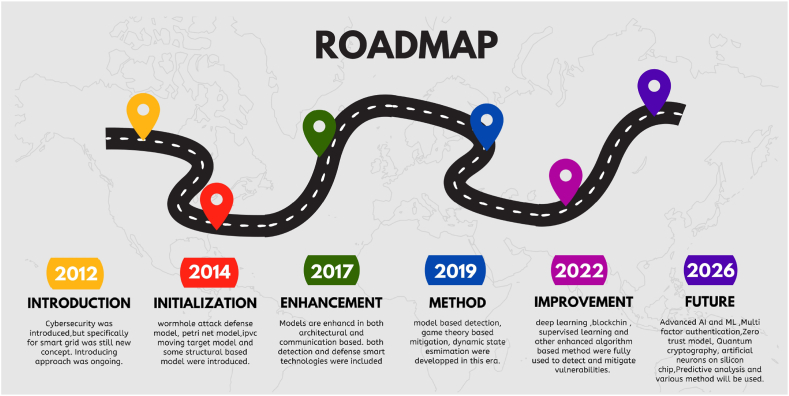
Future direction.

A smart grid represents a modernized electrical system that integrates advanced information and communication technologies to enhance the efficiency and reliability of power distribution. The extent of connectivity and reliance of the smart grid on the internet and other communication networks directly correlates with its vulnerability to potential attacks. Fig. 14 outlines how future cybersecurity for smart grids will be ensured through various technologies currently under research and implementation.

Blockchain, a distributed and immutable ledger, offers a transparent and secure way to store information about transactions. In the context of smart grids, it can be applied to ensure safe data flow across different nodes and reduce the possibility of cyberattacks. Additionally, AI algorithms can be used to identify irregularities in the system and alert operators to any security breaches.

IoT devices can monitor for cyberattacks and collect data from the grid. IoT sensors have the capacity to recognize changes in the system and notify operators of any hazards. Predictive analytics may be used to identify trends and patterns in a system and foresee possible online dangers. This technology enables operators to prevent intrusions before they begin. Multi-factor authentication requires users to provide various forms of identity as a security measure before they can access a system. With this technology, the smart grid can be secured so that only authorized individuals can access it.

Securing the future smart grid requires a comprehensive approach, integrating various advanced technologies to address the dynamic landscape of cyber threats. Blockchain technology, with its decentralized and immutable ledger, stands as a foundational element, ensuring the integrity of data and transactions within the smart grid. This not only provides a secure means of storing information but also establishes transparency in data transfer, reducing the susceptibility to cyberattacks.

Artificial Intelligence (AI) plays a pivotal role in enhancing the smart grid's cybersecurity posture. AI algorithms are adept at swiftly identifying anomalies and potential security breaches within the system. By continuously analyzing data, AI systems can detect unusual patterns or behaviors, enabling quick responses to mitigate emerging threats. This dynamic threat detection capability significantly enhances the resilience of the smart grid against a wide array of cyber risks.

The Internet of Things (IoT) is another crucial component in fortifying the smart grid's defenses. IoT devices, strategically deployed throughout the grid infrastructure, act as vigilant sensors, collecting real-time data on grid performance. These devices can identify system changes and promptly alert operators to potential cyber threats. The seamless integration of IoT technology thus provides enhanced situational awareness, enabling proactive measures to safeguard against unforeseen risks.

Predictive analytics is a proactive method for foreseeing and averting possible cyber risks. Predictive analytics forecasts future cyber risks before they occur by studying past data and finding trends. By enabling operators to take preemptive action, this proactive strategy improves the overall security posture of the smart grid.

In addition to these technologies, multi-factor authentication serves as a robust access control mechanism. This ensures that only authorized users with verified identities can access the smart grid, thereby reducing the risk of unauthorized access and potential security breaches.

By embracing a multifaceted approach that incorporates blockchain, AI, IoT, predictive analytics, and robust access control measures, the future smart grid can establish a resilient cybersecurity framework. This amalgamation of technologies not only addresses current vulnerabilities but also prepares the smart grid for emerging threats, ensuring the reliable and secure operation of critical infrastructure.

In conclusion, modern technology will be required for the smart grid of the future to ensure cybersecurity. Several technologies, including blockchain, AI, IoT, predictive analytics, and multi-factor authentication, will be utilized to safeguard the smart grid from online dangers.

## 11 Conclusion

Ensuring the security of smart grid networks is crucial and plays an essential role in facilitating the widespread adoption of smart grid technologies. Previous studies have highlighted a limited focus on assessing cybersecurity options for smart grid networks. This article aims to address the gaps in prior research by providing a comprehensive analysis of potential attacks on smart grids and a comparative evaluation of security approaches. In this work, we propose a comparison of the integrity, availability, confidentiality, and impact of cyberattacks. Additionally, we introduce a new classification of cyber attacks and broadly describe their detection and defense techniques. However, a key limitation of this paper lies in its broad characterization of cybersecurity options and the comparative evaluation of security approaches for smart grid networks. While advocating for innovative approaches, the paper lacks specificity in addressing the nuanced strengths and weaknesses of existing strategies. This limits the depth of proposed solutions, suggesting the need for future research to delve into the practical implementation and effectiveness of cybersecurity measures. This study underscores the necessity for innovative approaches that comprehensively address security concerns in smart grid infrastructures while maintaining operational efficiency and reliability.

## Funding statement

This research did not receive any specific grant from funding agencies in the public, commercial, or not-for-profit sectors.

## Data availability

This study does not involve the use of data. The research was conducted through literature review and analysis of existing papers.

## Additional information

No additional information is available for this paper.

## CRediT authorship contribution statement

**Bishowjit Paul:** Writing – review & editing, Writing – original draft, Visualization, Validation, Software, Resources, Project administration, Methodology, Investigation, Funding acquisition, Formal analysis, Data curation, Conceptualization. **Auvizit Sarker:** Writing – review & editing, Writing – original draft, Visualization, Validation, Software, Resources, Project administration, Methodology, Investigation, Formal analysis, Data curation, Conceptualization. **Sarafat Hussain Abhi:** Supervision. **Sajal Kumar Das:** Supervision. **Md. Firoj Ali:** Supervision, Dr. **Md Manirul Islam:** Supervision. **Md. Robiul Islam:** Supervision. **Sumaya Ishrat Moyeen:** Supervision. **Md. Faisal Rahman Badal:** Supervision. **Md. Hafiz Ahamed:** Supervision. **Subrata Kumar Sarker:** Supervision. **Prangon Das:** Supervision. **Md. Mehedi Hasan:** Supervision, Software. **Nazmus Saqib:** Supervision.

## Declaration of competing interest

The authors declare the following financial interests/personal relationships which may be considered as potential competing interests: Bishowjit Paul reports administrative support, statistical analysis, and travel were provided by 10.13039/501100016173Rajshahi University of Engineering and Technology, Rajshahi, Bangladesh. Bishowjit Paul reports a relationship with 10.13039/501100016173Rajshahi University of Engineering and Technology that includes: non-financial support. In compliance with ethical standards, I confirm that I have no other relationships, activities, or affiliations that could be interpreted as a conflict of interest by the reader. I am not serving in any editorial capacity for the journal to which this manuscript is being submitted.

Bishowjit Paul, Rajshahi University of Engineering & Technology, Rajshahi, Bangladesh. If there are other authors, they declare that they have no known competing financial interests or personal relationships that could have appeared to influence the work reported in this paper.
